# Aggregated LDL turn human macrophages into foam cells and induce mitochondrial dysfunction without triggering oxidative or endoplasmic reticulum stress

**DOI:** 10.1371/journal.pone.0245797

**Published:** 2021-01-25

**Authors:** Gabriela M. Sanda, Camelia S. Stancu, Mariana Deleanu, Laura Toma, Loredan S. Niculescu, Anca V. Sima

**Affiliations:** 1 Lipidomics Department, Institute of Cellular Biology and Pathology “Nicolae Simionescu” of the Romanian Academy, Bucharest, Romania; 2 Faculty of Biotechnology, University of Agronomical Sciences and Veterinary Medicine, Bucharest, Romania; Xiangtan University, CHINA

## Abstract

Uptake of modified lipoproteins by macrophages turns them into foam cells, the hallmark of the atherosclerotic plaque. The initiation and progression of atherosclerosis have been associated with mitochondrial dysfunction. It is known that aggregated low-density lipoproteins (agLDL) induce massive cholesterol accumulation in macrophages in contrast with native LDL (nLDL) and oxidized LDL (oxLDL). In the present study we aimed to assess the effect of agLDL on the mitochondria and ER function in macrophage-derived foam cells, in an attempt to estimate the potential of these cells, known constituents of early fatty streaks, to generate atheroma in the absence of oxidative stress. Results show that agLDL induce excessive accumulation of free (FC) and esterified cholesterol in THP-1 macrophages and determine mitochondrial dysfunction expressed as decreased mitochondrial membrane potential and diminished intracellular ATP levels, without generating mitochondrial reactive oxygen species (ROS) production. AgLDL did not stimulate intracellular ROS (superoxide anion or hydrogen peroxide) production, and did not trigger endoplasmic reticulum stress (ERS) or apoptosis. In contrast to agLDL, oxLDL did not modify FC levels, but stimulated the accumulation of 7-ketocholesterol in the cells, generating oxidative stress which is associated with an increased mitochondrial dysfunction, ERS and apoptosis. Taken together, our results reveal that agLDL induce foam cells formation and mild mitochondrial dysfunction in human macrophages without triggering oxidative or ERS. These data could partially explain the early formation of fatty streaks in the intima of human arteries by interaction of monocyte-derived macrophages with non-oxidatively aggregated LDL generating foam cells, which cannot evolve into atherosclerotic plaques in the absence of the oxidative stress.

## Introduction

The accumulation of macrophage-derived foam cells in the arterial intima is the hallmark of the early-stage atherosclerosis [1–3]. Intimal foam cells develop mainly from monocyte-derived macrophages, due to the continuous uptake of modified low-density lipoproteins (LDL) trapped in the sub-endothelium [4,5]. Aggregation of LDL is a structural, non-oxidative alteration happening in the intima of the arterial wall, many LDL particles being associated with the extracellular matrix proteoglycans in the arterial intima [6,7]. Aggregated LDL (agLDL) determine massive free (FC) and esterified (EC) cholesterol accumulation in macrophages [8–10]. AgLDL can be internalized by LDL receptor-related protein (LRP1) [11], by a phagocytic process [8], or by sequestration in the macrophages surface-connected compartments and plasma membrane invaginations by a process named patocytosis [9,10].

In contrast with native LDL (nLDL) or agLDL, oxidized LDL contain 7-ketocholesterol (7-KC), the most prominent oxysterol formed during the oxidative modification of LDL [12]. Previous data, as well our own, show that oxLDL induce the intracellular accumulation of 7-KC in macrophages, triggering oxidative and endoplasmic reticulum stress (ERS), mitochondrial dysfunction and apoptosis [12–15].

It is known that exposure of macrophages to agLDL determines the accumulation of FC in the late endosome/lysosomes [16,17]. FC can be transported from the late endosomes to other cellular organelles such as mitochondria and endoplasmic reticulum (ER), where the excessive concentrations of FC can affect the function of these cellular compartments. If such dysregulation of the lipid metabolism persists and combines with other pro-oxidant insults, it may lead to ER stress (ERS). Ultimately, this can result in apoptotic cell death [18,19]. The main sources of intracellular oxidative stress in macrophages are mitochondria and NADPH oxidase family [20].

In mitochondria, reactive oxygen species (ROS) are formed as by-products of the oxidative phosphorylation that leads to ATP production. Mitochondrial functions are altered by severe and/or prolonged oxidative stress [21]. The depolarization of mitochondrial membrane potential (ΔΨm), the driving force for mitochondrial ATP synthesis, causes mitochondrial dysfunction known to be associated with the initiation and progression of atherosclerosis [22,23].

ER is the organelle involved in the folding and trafficking of proteins, having an important role in maintaining the cellular homeostasis. Different studies show an interdependence between ROS and ERS [24].

Data regarding the agLDL specific effects on mitochondria and ER from macrophages are lacking, the existing studies being focused mainly on the mechanisms of internalization, catabolism and lipid loading. Thus, the present paper aims to evaluate the effects of agLDL on the function of mitochondria and ER in macrophage-derived foam cells, questioning if non-oxidatively modified LDL could play a role in atherosclerosis inception. The results were compared to those obtained for nLDL and oxLDL effects in macrophages in an attempt to estimate the potential of macrophage-derived foam cells, known constituents of early fatty streaks, to generate atheroma in the absence of oxidative stress.

## Materials and methods

### Reagents

RPMI-1640 medium, penicillin, streptomycin, phorbol-12-myristate-13-acetate (PMA), Oil Red O, protease inhibitor cocktail, sodium orthovanadate, sodium fluoride, dihydroethidium (DHE), 2',7'-dichlorofluorescein diacetate (DCFH-DA) and thiazolyl blue tetrazolium bromide (MTT) were purchased from Sigma-Aldrich, St. Louis, MO, USA. Fetal calf serum was from EuroClone, Siziano, Italy. TRIzol reagent and SYBR Select master mix were from Applied Biosystems, CA, USA. High Capacity cDNA Reverse Transcription kit, MitoSOX^TM^ Red mitochondrial superoxide indicator (Invitrogen) and JC-1 (5,5’,6,6’-tetrachloro-1,1’,3,3’-tetraethylbenzimidazolylcarbocyanine iodide) dye (Molecular Probes) were from Thermo Fisher Scientific, Waltham, MA, USA.

Primary antibodies against human LRP1 (ab20753, mouse monoclonal, 1:1000), CD36 (ab36977, rabbit polyclonal, 1:1000), phospho- inositol-requiring transmembrane kinase/endoribonuclease 1α (p-IRE1; Ser724, ab124945, rabbit monoclonal, 1:1000), CCAAT/-enhancer-binding protein homologous protein (CHOP/DDIT3; ab11419, mouse monoclonal, 1:1000) and horseradish peroxidase-conjugated goat secondary antibodies against mouse or rabbit IgG (1:10000) were obtained from Abcam, Cambridge, UK. The antibodies to human phospho-eukaryotic Initiation Factor 2α (p-eIF2α; Ser52, sc-101670, rabbit polyclonal, 1:500), eIF2α (sc-11386, rabbit polyclonal, 1:500), IRE1α (sc-20790, rabbit polyconal, 1:800), activating transcription factor 6α (ATF-6α; sc-166659, mouse monoclonal, 1:400), glucose-regulated protein 78 (Grp78; KDEL ER Marker, sc-58774, mouse monoclonal, 1:800) and β-actin (sc-47778, mouse monoclonal, 1:4000) were purchased from Santa Cruz Biotechnology, Santa Cruz, CA, USA.

### LDL isolation and modification

Native LDL (nLDL) were isolated by density gradient ultracentrifugation from human plasma of healthy donors, as previously described [25]. AgLDL were obtained according to previous published data [26,27]. In brief, nLDL (1 mg/mL) were vortexed for 4 min at room temperature at maximal speed. Modified LDL were then centrifuged at 10,000xg for 10 min and the precipitated fraction composed of 100% agLDL was added to cell cultures [26,27]. *In vitro* oxLDL was prepared by incubating nLDL with 10 μM CuSO_4_ for 24 h at 37°C in the absence of antioxidant protection, and characterized as described [25,28]. The lipid peroxides levels in LDL were measured as thiobarbituric acid reactive substances (TBARS) by Ultra High Pressure Liquid Chromatography (UHPLC) method [29] and expressed as malondialdehyde (MDA) pmoles/mg LDL protein.

### Cell culture and experimental procedure

Human leukemic monocytic THP-1 cells (ATCC, Manassas, VA, USA) were differentiated into macrophages as previously described [12]. Macrophages were incubated in serum free-medium for 24 h with nLDL, agLDL or oxLDL (100 μg protein/ml). Macrophages incubated in similar conditions, but not exposed to LDL were considered control cells. After LDL incubation, cells were exhaustively washed as in [30] and collected for either mRNA/protein or lipid extraction.

### Lipid staining with Oil Red O

To evidence the lipid droplets accumulated in agLDL-exposed macrophages, Oil Red O staining was performed. The cells plated on coverslips placed in 24 well plates (5x10^5^ cells/well) were incubated for 24 h with agLDL, oxLDL or nLDL (100 μg protein/ml). After washing with phosphate buffer saline (PBS), the cells were fixed in 4% paraformaldehyde (10 min), washed twice with PBS, stained with Oil Red O (0.3% in 60% isopropanol, 15 min) to evidence the neutral lipids and examined by light microscopy with a Microphot FXA Nikon microscope using filter block B-2A.

### Analysis of intracellular cholesterol accumulation

Free cholesterol (FC) and total cholesterol (TC) content of nLDL, agLDL and oxLDL, and of the macrophages exposed to nLDL, agLDL or oxLDL were analyzed by gas chromatography–mass spectrometry (GC-MS), as previously reported [28].

### RNA extraction and real-time PCR analysis

Total RNA was isolated from cultured macrophages using TRIzol reagent following the manufacturer’s protocol. One μg of total RNA was reverse transcribed into cDNA using the High Capacity cDNA Reverse Transcription kit. cDNA was analyzed by real-time PCR using the ViiA7 real-time PCR system (Applied Biosystems, Life Technologies, Carlsbad, USA) and SYBR Select master mix. The human specific primers for studied genes are listed in Table 1. The relative quantification was performed using the Fit-Point method [31].

**Table 1:** Primers used for real-time PCR analysis.

Gene	GeneBank accession number	Sequences of oligonucleotide primers	Amplicon size (bp)
**LRP1**	NM_002332.3	FW: 5'- GAGCTGAACCACGCCTTTG-3'	79
		RW: 5'- GGTAGACACTGCCACTCCGATAC-3'	
**CD36**	NM_001001548.2	FW: 5'-TCTTTCCTGCAGCCCAATG-3'	60
		RW: 5'-AGCCTCTGTTCCAACTGATAGTGA-3'	
**Bax**	NM_001291428.1	FW: 5'- CCTGGACCCGGTGCCTCAGG-3'	245
		RW: 5'- TGGTGCACAGGGCCTTGAGC-3'	
**Bcl-2**	NM_000633.2	FW: 5'- AGGGCAGTGTGGTCTCCGAATG-3'	249
		RW: 5'- CCATTGCCTCTCCTCACGTTCC-3'	
**β-actin**	NM_001101.3	FW: 5'-GTCTTCCCCTCCATCGT -3'	82
		RW: 5'-CGTCGCCCACATAGGAAT -3'	

LRP1, LDL receptor-related protein; CD36, scavenger receptor CD36; Bax, Bcl-2 associated X; Bcl-2, B-cell lymphoma 2.

### Immunoblot assay

Western Blot analysis was performed as previously described [12].

### The quantification of ROS production

Mitochondrial reactive oxygen species ROS (mtROS) generation was measured using MitoSOX^TM^ Red mitochondrial superoxide indicator according to the manufacturer instructions and expressed as relative arbitrary units per total cell protein. Intracellular ROS levels in cultured macrophages were measured using DHE fluorescent marker, which especially detects the superoxide anion (O_2_^-^) [12] or DCFH-DA, probably the most commonly used probe for measuring cellular hydrogen peroxide (H_2_O_2_) [32]. The results were expressed as relative fluorescence units per total cell protein.

The NADPH oxidase (NADPHox) activity in whole cells was estimated by using a lucigenin-enhanced chemiluminescence assay as previously described [25] and expressed as relative light units per total cell protein.

The lipid peroxides levels in the culture media were measured as TBARS by UHPLC method described in [33] and expressed as MDA pmoles/ml.

### Evaluation of the mitochondrial membrane potential

JC-1 dye was used to assess the mitochondrial membrane potential (ΔΨm) as described in [34]. In brief, after LDL exposure, macrophages seeded on 12-well plates were loaded with JC-1 dye (5 μg/ml, 10 min, 37°C). The JC-1 in excess was removed by extensive washing, then the cells were scraped and the fluorescence intensity was measured using a spectrofluorometer Tecan Infinite M200 (Tecan Austria, Austria) (excitation 485 nm and 500 nm; emission 535 nm and 600 nm).

### Measurement of ATP content

The detection of the cellular ATP was done with ViaLight™ plus kit (Lonza, Walkersville, MD, USA), according to manufacturer’s instructions.

### Evaluation of cell viability

The cell viability was measured using the tetrazolium salt (MTT) and CytoTox-ONE™ Homogeneous Membrane Integrity Assay (Promega, Madison, USA), according to manufacturer instructions. In addition, Hoechst 33258 was used to estimate the proliferation rate by measuring the cellular DNA content [25].

### Statistical analysis

The statistical analysis of the experimental data was done using the dedicated SPSS software (IBM SPSS v21, IBM Ireland, Dublin, Ireland). The Mann-Whitney (U-test) and T-test were used for the inter-group validation of the obtained results. One-way ANOVA test was used to compare differences between the experimental groups (the control cells, nLDL, agLDL and oxLDL-exposed macrophages). P < 0.05 values were considered statistically significant. Data were expressed as mean ± standard deviation (SD) and are representative for at least three independent experiments.

## Results

### AgLDL induce excessive intracellular cholesterol accumulation in THP-1 macrophages

**Fig 1:**
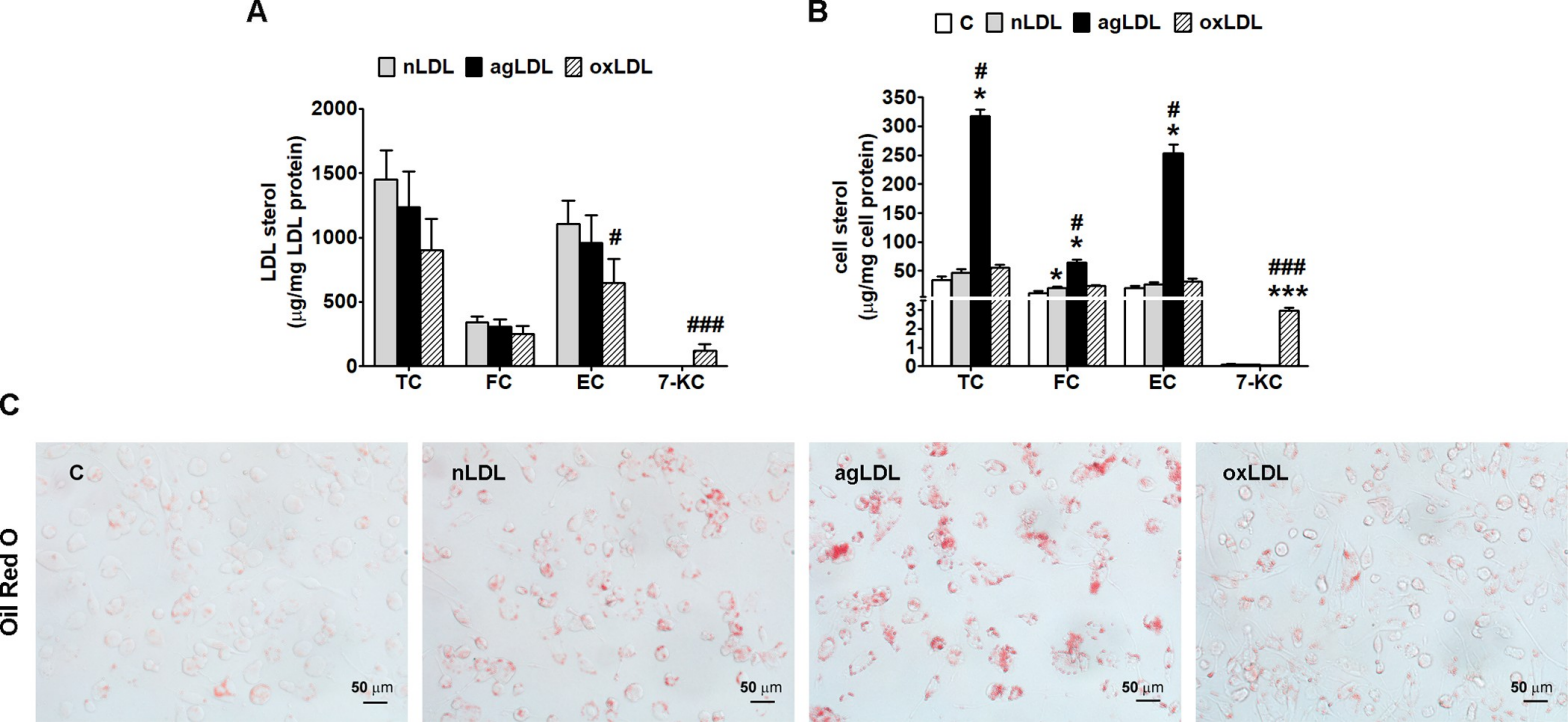
AgLDL induce intracellular cholesterol accumulation in THP-1 macrophages. Cells were exposed for 24 h to 100 μg/ml aggregated LDL (agLDL), oxidized (oxLDL) or native LDL (nLDL). Cells incubated in the same conditions without LDL were considered control cells (C). (A) Total (TC), free (FC) and esterified cholesterol (EC) and 7-ketocholesterol (7-KC) levels in agLDL, oxLDL and nLDL analyzed by GC-MS and expressed as LDL sterol relative to LDL protein. (B) Intracellular TC, FC, EC and 7-KC levels analyzed by GC-MS and expressed as cell sterol relative to total cell protein. (C) Oil Red O staining showing neutral lipid accumulation, bar = 50 μm. All data are expressed as fold change versus C and presented as mean ± SD (n = 4). *p < 0.05, ***p < 0.001 vs. C; ^#^p < 0.05, ^###^p < 0.001 vs. nLDL.

AgLDL prepared *in vitro* have similar lipid peroxides levels as nLDL (0.049 ± 0.017 nmoles MDA/mg protein) and the same FC/protein and EC/protein ratios as nLDL (μg sterol/mg protein) (Fig 1A). OxLDL have significantly increased lipid peroxides levels (1.05 ± 0.44 nmoles MDA/mg protein, p < 0.001 vs. nLDL), are characterized by the presence of 7-ketocholesterol (7-KC) (121.90 ± 40.17μg sterol/mg protein, p < 0.001 vs. nLDL) and a significantly reduced EC content (41%, p < 0.05 vs. nLDL) (Fig 1A).

The incubation of macrophages with agLDL, induced a significant increase of the cellular levels of TC, FC and EC as compared to C, nLDL or oxLDL (p < 10.001 for all studied groups by ANOVA). AgLDL significantly increased the cellular levels of TC (9.3-fold, p = 0.021 vs. C and 6.8-fold, p = 0.021 vs. nLDL), FC (5.4-fold, p = 0.021 vs. C and 3.2-fold, p = 0.021 vs. nLDL) and EC (12.5-fold, p = 0.021 vs. C and 9.7-fold, p = 0.021 vs. nLDL (Fig 1B). The incubation of macrophages with nLDL, as compared to C, significantly increased the cellular levels of FC (70%, p = 0.021). Moreover, oxLDL did not change FC and EC levels, but induced 7-KC accumulation in macrophages (Fig 1B).

To visualize the lipids accumulated in THP-1 macrophages following the exposure to modified LDL or nLDL, the cells were stained with Oil Red O. Light microscopy images revealed a considerably increased number of lipid droplets in cells incubated with agLDL compared with cells incubated with oxLDL or nLDL (Fig 1C).

**Fig 2:**
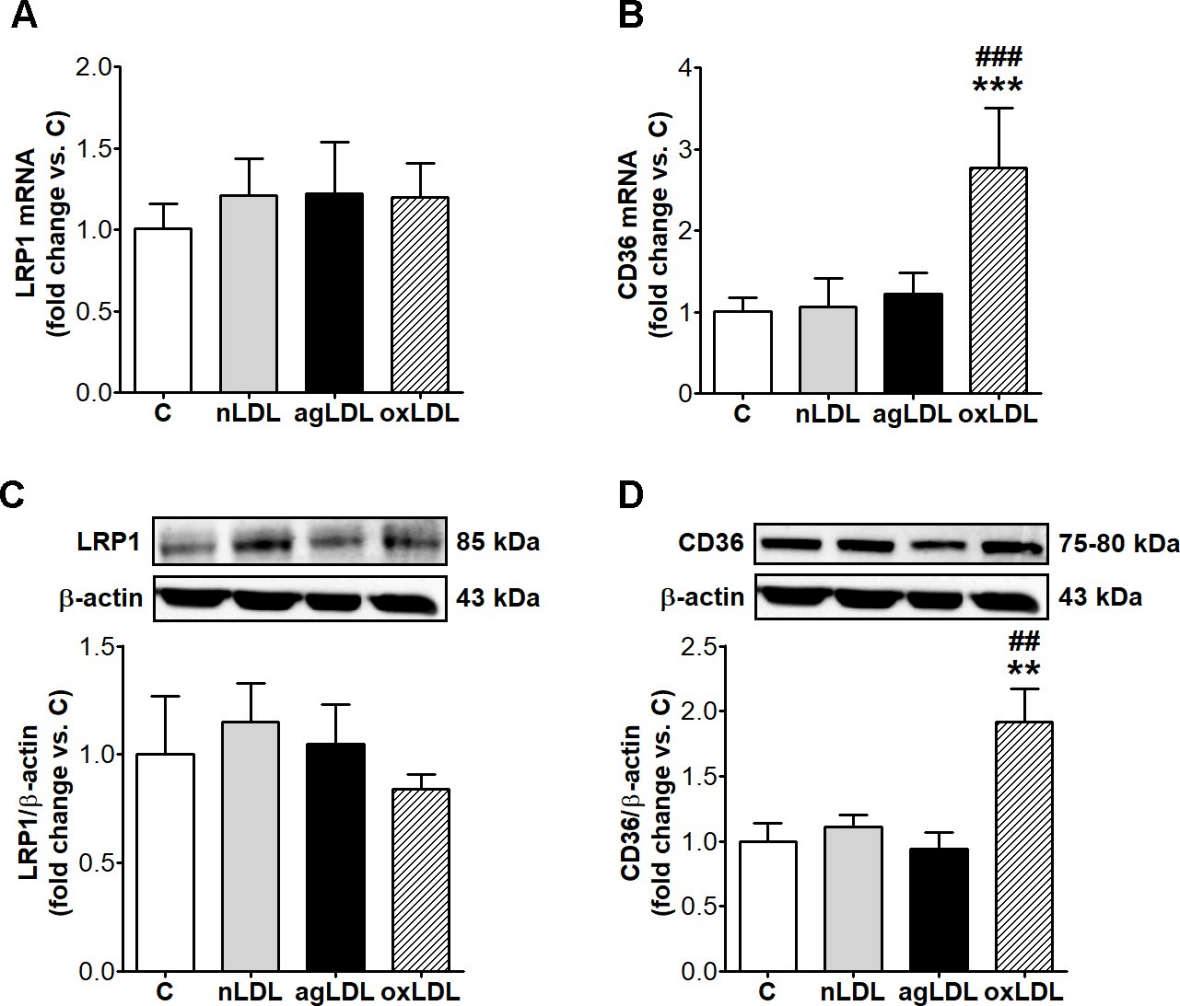
AgLDL do not modify LRP1 and CD36 expression in THP-1 macrophages. Cells were exposed for 24 h to 100 μg/ml aggregated LDL (agLDL), oxidized LDL (oxLDL) or native LDL (nLDL). Cells incubated in the same conditions without LDL were considered control cells (C). mRNA levels of LRP1 (A) and CD36 (B) relative to β-actin (n = 6). Representative blot and densitometric analysis of LRP1 (C) and CD36 (D) protein normalized to β-actin (n = 4). All data are represented as fold change versus C and presented as mean ± SD. **p < 0.01, ***p < 0.001 vs. C; ^##^p < 0.01, ^###^p < 0.001 vs. nLDL.

The incubation of macrophages with agLDL, compared to C or nLDL, did not change LRP1 and CD36 gene and protein expression. OxLDL did not alter LRP1 expression, but significantly increased CD36 gene and protein expression compared to nLDL (2.6-fold, p < 0.001 and 74%, p < 0.01; p < 0.001 for all studied groups by ANOVA) (Fig 2).

### AgLDL induce mitochondrial dysfunction

**Fig 3:**
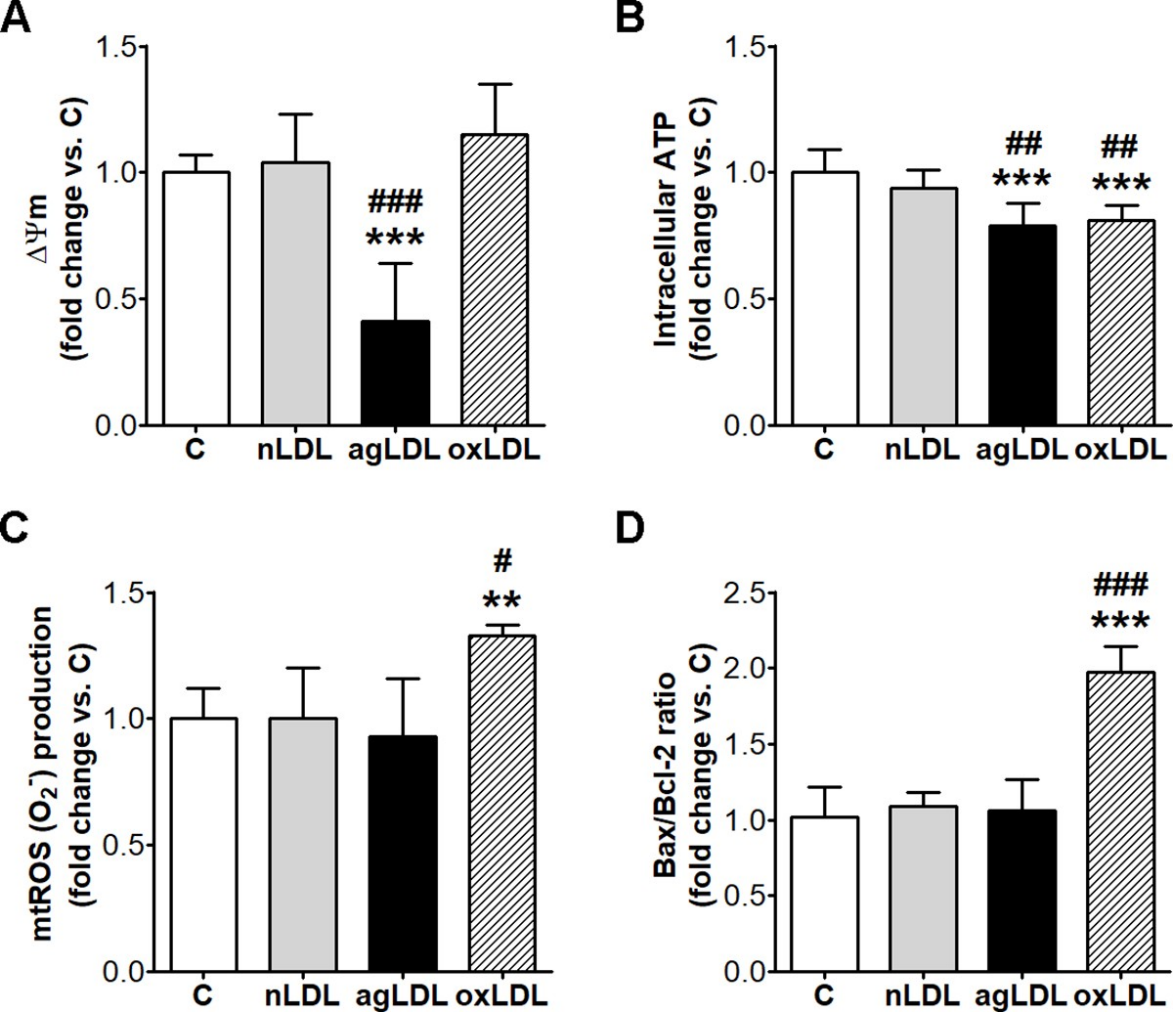
AgLDL induce the decrease of ΔΨm and ATP in THP-1 macrophages, without mtROS or apoptosis. Cells were exposed for 24 h to 100 μg/ml aggregated LDL (agLDL), oxidized (oxLDL) or native LDL (nLDL). Cells incubated in the same conditions without LDL were considered control cells (C). (A) Mitochondrial membrane potential (ΔΨm) expressed as JC-1 red/green fluorescence intensity ratio (n = 6). (B) Intracelullar ATP levels assessed using ViaLight™ plus kit and expressed as relative luminescence units per total cell protein (n = 5). (C) Mitochondrial ROS (mtROS) assessed using MitoSOX^TM^ Red mitochondrial superoxide indicator and expressed as relative arbitrary units per total cell protein (n = 4). (D) Bax and Bcl-2 mRNA levels relative to β-actin assessed by real-time PCR and expressed as Bax/Bcl-2 ratio (n = 4). All data are expressed as fold change versus C and presented as mean ± SD. **p < 0.01, ***p < 0.001 vs. C; ^#^p < 0.05, ^##^p < 0.01, ^###^p < 0.001 vs. nLDL.

The effect of lipid loading on mitochondrial function in macrophages was assessed. The experiments showed that, compared to C or nLDL, agLDL significantly decreased ΔΨm (59%, p < 0.001 vs. C and 61%, p < 0.001 vs. nLDL; p < 0.001 for all studied groups by ANOVA) and intracellular ATP levels (21%, p < 0.001 vs. C and 16%, p = 0.003 vs. nLDL; p < 0.001 for all studied groups by ANOVA) (Fig 3A and 3B), which indicates an impaired mitochondrial function. Interestingly, agLDL did not stimulate mtROS production (Fig 3C). Moreover, agLDL did not induce apoptosis, Bax/Bcl-2 ratio remaining unaltered (Fig 3D).

The exposure of macrophages to oxLDL did not affect ΔΨm, but significantly decreased intracellular ATP levels (19%, p < 0.001 vs. C and 13%, p < 0.01 vs. nLDL; p < 0.001 for all studied groups by ANOVA). Furthermore, oxLDL stimulated mtROS production (33%, p < 0.01 vs. C and 33%, p < 0.05 vs. nLDL; p = 0.056 for all studied groups by ANOVA) and increased Bax/Bcl-2 ratio (94%, p < 0.001 vs. C and 80%, p < 0.001 vs. nLDL; p < 0.001 for all studied groups by ANOVA) (Fig 3).

### AgLDL do not stimulate intracellular ROS production

**Fig 4:**
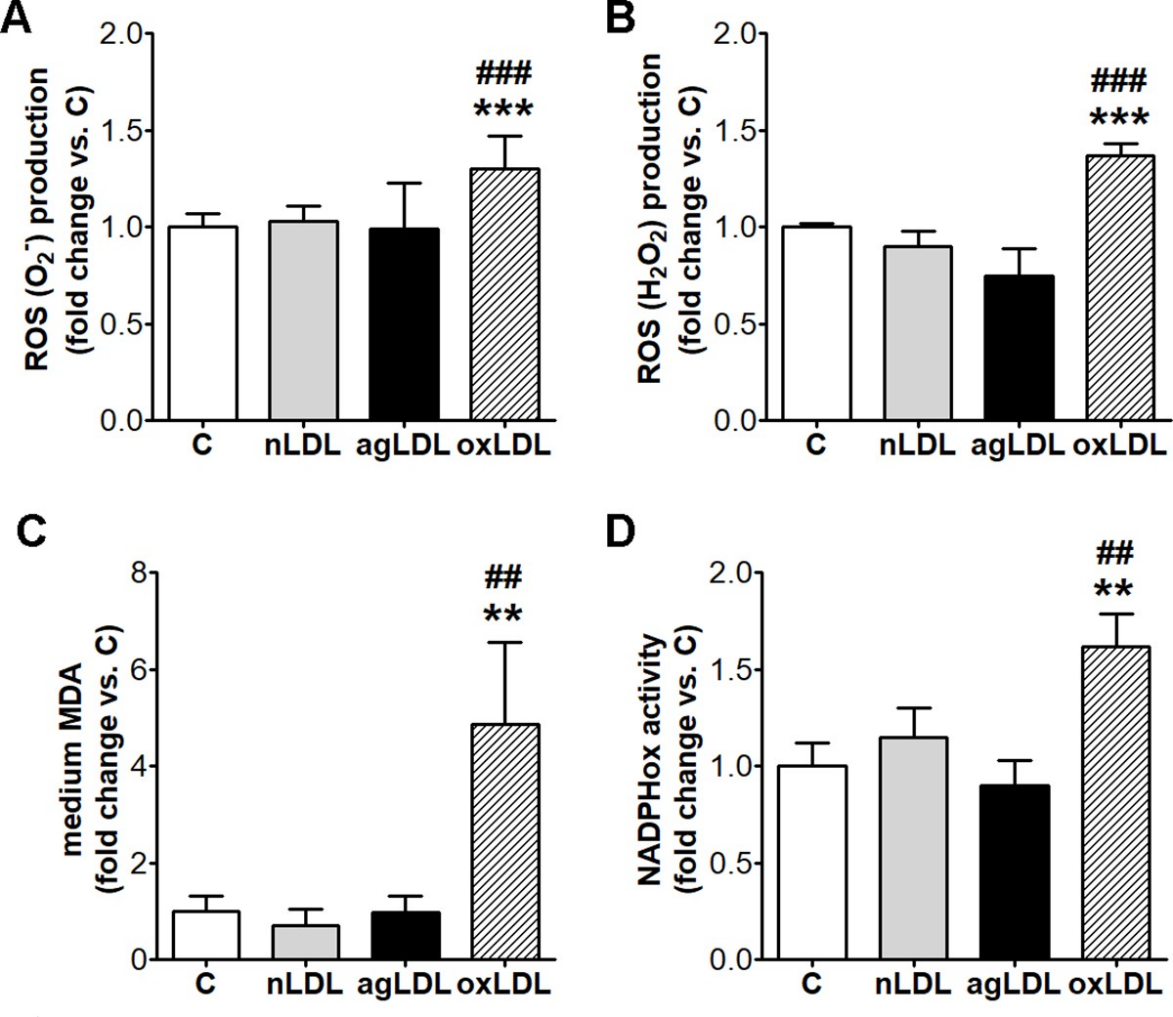
AgLDL do not induce oxidative stress in THP-1 macrophages. Cells were exposed for 24 h to 100 μg/ml aggregated (agLDL), oxidized LDL (oxLDL) or native LDL (nLDL). Cells incubated in the same conditions without LDL were considered control cells (C). Intracellular levels of superoxide (O_2_^-^) (n = 6) (A) and of hydrogen peroxide (H_2_O_2_) (n = 4) (B) assessed using dihydroethidium (DHE) and 2',7'-dichlorofluorescein diacetate (DCFH-DA), respectively, and expressed as relative fluorescence units per total cell protein. (C) Lipid peroxides levels in the culture media assessed as TBARS by UHPLC and expressed as malondialdehyde (MDA) pmoles/ml (n = 5). (D) NADPH oxidase (NADPHox) activity assessed using a lucigenin-enhanced chemiluminescence assay and expressed as relative luminescence units per total cell protein (n = 4). All data are expressed as fold change versus C and presented as mean ± SD. **p < 0.01, ***p < 0.001 vs. C; ^##^p < 0.01, ^###^p < 0.001 vs. nLDL.

We questioned if, in our experimental conditions, agLDL may induce ROS production in macrophages. Results showed that agLDL- exposure of macrophages did not stimulate the intracellular ROS (O_2_^-^ or H_2_O_2_) production, and did not induce the presence of lipid peroxides in the culture medium (Fig 4A–4C). NADPHox activity was not increased by agLDL compared to nLDL (Fig 4D).

The incubation of macrophages with oxLDL, as compared with nLDL, stimulated intracellular ROS production (O_2_^-^, 26%, p < 0.001 and H_2_O_2_, 53%, p < 0.001; p < 0.001 for all studied groups by ANOVA), increased NADPHox activity (40%, p < 0.01; p < 0.001 for all studied groups by ANOVA) and the release of lipids peroxides in the culture medium (6.8-fold, p < 0.01; p < 0.001 for all studied groups by ANOVA) (Fig 4).

### AgLDL do not induce ER stress in macrophages

**Fig 5:**
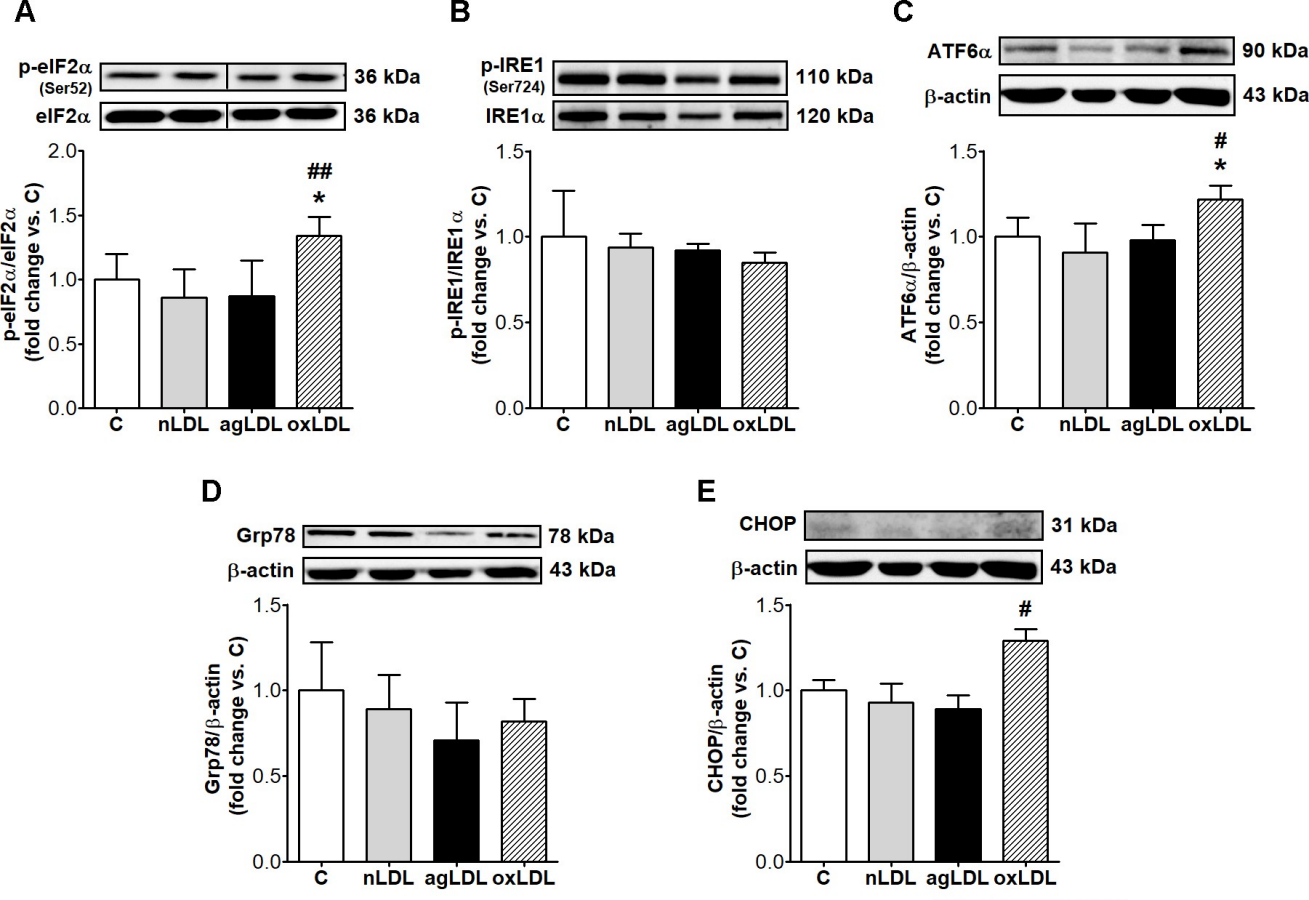
AgLDL do not trigger ER stress in THP-1 macrophages. Cells were exposed for 24 h to 100 μg/ml aggregated LDL (agLDL), oxidized LDL (oxLDL) or native LDL (nLDL). Cells incubated in the same conditions without LDL were considered control cells (C). Representative blots and densitometric analysis of phospho-eIF2α (p-eIF2α) normalized to total eIF2α (t-eIF2α) (n = 3) (A), phospho-IRE1 (p-IRE1) normalized to IRE1α (n = 4) (B), ATF6α (n = 4) (D), Grp78 (n = 3) (E) and CHOP (n = 4) (F) protein normalized to β-actin. All data are expressed as fold change versus C and presented as mean ± SD. *p < 0.05 vs. C; ^#^p < 0.05, ^##^p < 0.01 vs. nLDL.

Since our data showed that agLDL have induced excessive intracellular accumulation of FC, we questioned if agLDL could induce ERS in our experimental conditions. Exposure of macrophages to agLDL, compared to C or nLDL, did not stimulate the expression of any of the analyzed ER stress markers: the phosphorylation levels of eIF2α (phospho-eIF2α/total-eIF2α) and IRE1α (phospho-IRE1/total-IRE1α), and the protein expressions of ATF6α, Grp78 and CHOP (Fig 5).

The exposure of macrophages to oxLDL significantly increased the phosphorylation level of eIF2α (56%, p < 0.01 vs. nLDL; p = 0.001 for all studied groups by ANOVA), ATF6α (35%, p < 0.05 vs. nLDL; p = 0.015 for all studied groups by ANOVA) and CHOP (38%, p < 0.05 vs. nLDL; p < 0.001 for all studied groups by ANOVA) protein expression (Fig 5).

### AgLDL do not affect cell viability

**Fig 6:**
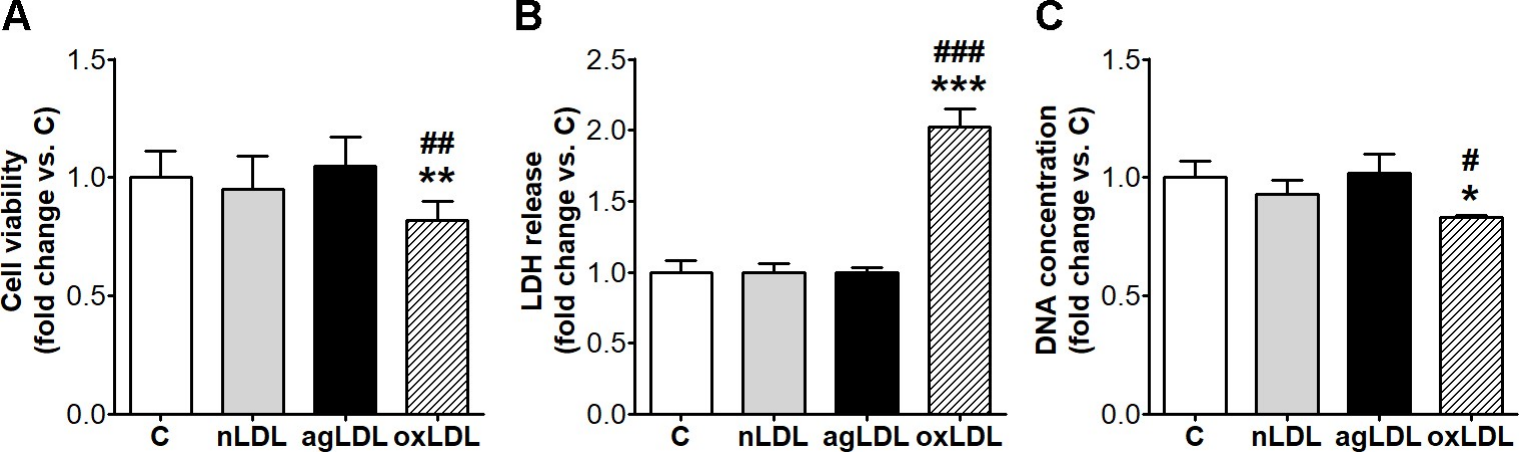
AgLDL do not affect viability of THP-1 macrophages. Cells were exposed for 24 h to 100 μg/ml aggregated LDL (agLDL), oxidized LDL (oxLDL) or native LDL (nLDL). Cells incubated in the same conditions without LDL were considered control cells (C). (A) Cell viability measured using an assay based on tetrazolium salt (MTT). (B) Lactate dehydrogenase (LDH) release in the culture medium assessed using CytoTox-ONE™ Homogeneous Membrane Integrity Assay. (C) Cell proliferation assessed by Hoechst 33258 fluorimetric assay, expressed as μg DNA. All data are represented as fold change versus C and presented as mean ± SD (n = 4). *p < 0.05, **p < 0.01, ***p < 0.001 vs. C; ^#^p < 0.05, ^##^p < 0.01, ^###^p < 0.001 vs. nLDL.

AgLDL did not alter cell viability, did not induce LDH release in the culture medium and did not affect cell proliferation (Fig 6). The incubation of macrophages with oxLDL significantly decreased cell viability (14%, p < 0.01 vs. nLDL; p = 0.001 for all studied groups by ANOVA), increased LDH release in the culture medium (2-fold, p < 0.001 vs. nLDL; p < 0.001 for all studied groups by ANOVA) and decreased cell proliferation (11%, p < 0.05 vs. nLDL; p = 0.011 for all studied groups by ANOVA) (Fig 6).

## Discussion

Macrophage-derived foam cells are constituents of the fatty streaks, the earliest and reversible atherogenic modification of the arterial wall [3]. Mitochondrial dysfunction is a key step that can lead the macrophages toward a pro-atherogenic state [21,23]. It is known that the exposure of macrophages to agLDL, which are non-oxidatively modified LDL, generates their transformation into foam cells. The novel finding of the present study is that these foam cells develop mitochondrial dysfunction, expressed by the reduced ΔΨm and intracellular ATP levels, without triggering ROS production (either in mitochondria, or in the cytoplasm) or ERS. Our results emphasize that in the absence of oxidative stressors, a structural, non-oxidative modification of LDL such as aggregation induces only a dysfunction in the energy metabolism of the cell, without stimulation of the oxidative stress.

LDL aggregation is an important non-oxidative modification undergoing in the vascular wall due to the trapping of the transcytosed LDL in the intima of the arterial wall, where they associate with the extracellular matrix proteoglycans [4,6]. Our data show that agLDL induce excessive intracellular accumulation of FC and EC in THP-1 macrophages and their transformation in foam cells, in good agreement with previous reports [16,30]. AgLDL did not change LRP1 and CD36 expression, in good agreement with previously published data [30]. OxLDL did not modify FC levels, but stimulated the accumulation of 7-KC in the cells, generating ERS [12].

Our results demonstrate for the first time that the exposure of macrophages to agLDL significantly decreases ΔΨm and as a consequence, the intracellular ATP levels, without stimulating oxidative stress (mtROS or intracellular NADPHox-derived ROS). Thus, we may presume that in our experimental conditions, agLDL induce mitochondrial dysfunction. It is known that production of ROS is associated with mitochondrial dysfunction and has been associated with the initiation and progression of the atherosclerotic plaque [23]. ROS play a very important role in the generation of mitochondrial dysfunction and trigger a vicious circle: ROS harm mitochondria, generating dysfunctional mitochondria, and dysfunctional mitochondria produce more ROS [35]. In contrast to agLDL, oxLDL increase the intracellular oxidative stress which is associated with an increased mitochondrial dysfunction, ERS and apoptosis. In good agreement with our findings, Zamora et al reported that the partial loss of mitochondrial function is not enough to promote pro-atherogenic activities in macrophages [36]. Therefore, it is a good reason to belive that the foam cells generated by agLDL-interaction with macrophages may be considered the equivalent of fatty streak. Moreover, agLDL-exposure of THP-1 macrophages did not induce apoptosis of the cells, Bax/Bcl-2 ratio being unaltered, in good agreement with previously published data [37]. In addition, we show for the first time that agLDL incubation did not trigger ERS in THP-1 macrophages and did not affect their viability and proliferation, probably due to the absence of oxidative stress which is a determinant of ERS [24]. A complementary explanation could be the interesting report that intracellular lipid droplets protect against ERS by acting like buffers that sequester the misfolded proteins and the excess lipids, rebalancing ER lipid homeostasis [38].

In conclusion, our results demonstrate that the FC loading induced by agLDL exposure in the absence of the oxidative stress is not sufficient to generate advanced mitochondrial dysfunction and ERS in foam cells-derived macrophages, ROS being probably the key player in this process.

These data could partially explain the early formation of fatty streaks in the intima of human arteries by the interaction of accumulated, non-oxidatively aggregated LDL with monocyte-derived macrophages generating foam cells, which cannot evolve into atherosclerotic plaque in the absence of the oxidative stress.

## Supporting information

S1 File (DOCX)
