# Supplementary material for: Aggregated LDL turn human macrophages into foam cells and induce mitochondrial dysfunction without triggering oxidative or endoplasmic reticulum stress
Source: PLoS One. 2021 Jan 25;16(1):e0245797. doi: 10.1371/journal.pone.0245797 (PMC7833132; doi:10.1371/journal.pone.0245797)
Supplement: S1 File — (DOCX) [file pone.0245797.s001.docx]

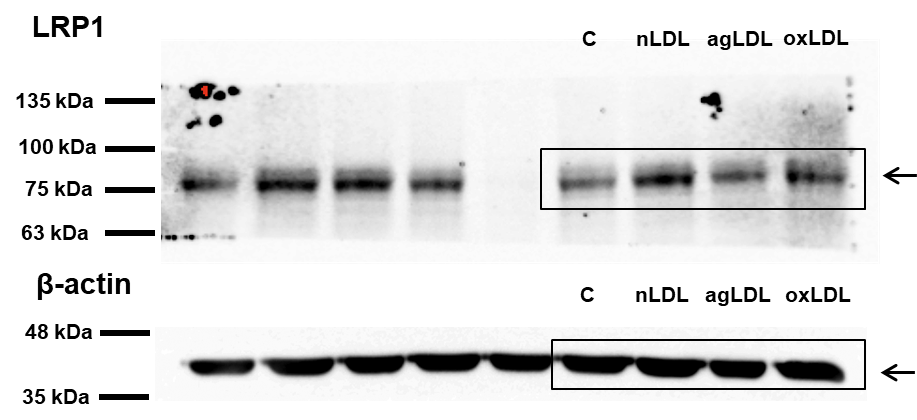


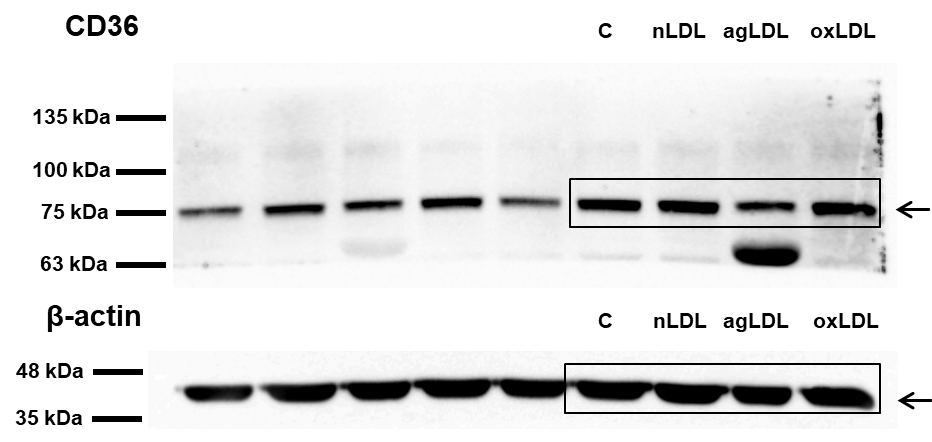


**Fig 2** raw: images acquired using ImageQuant LAS4000 imaging system and ImageQuant-TL software


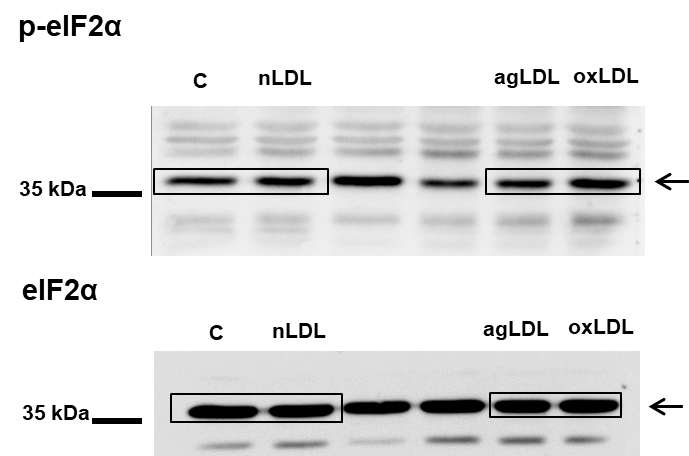


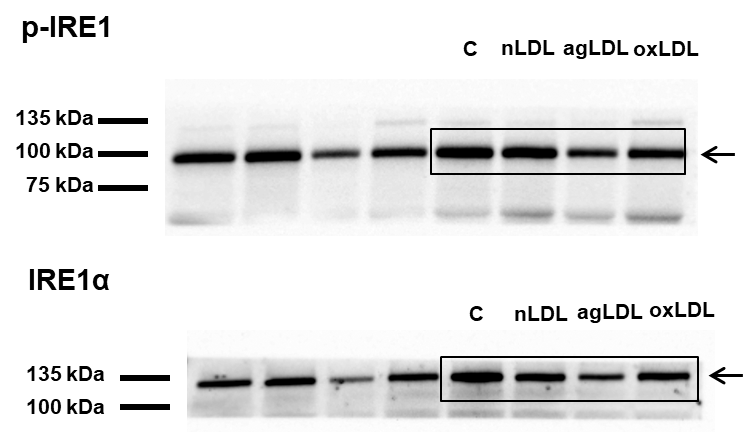


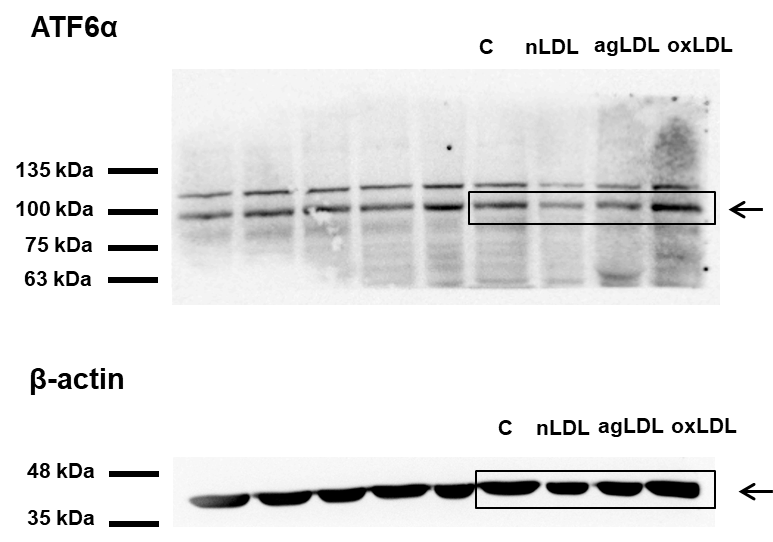


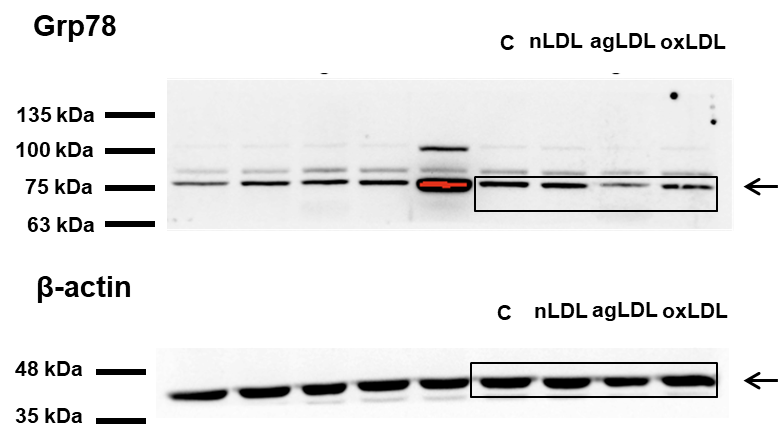


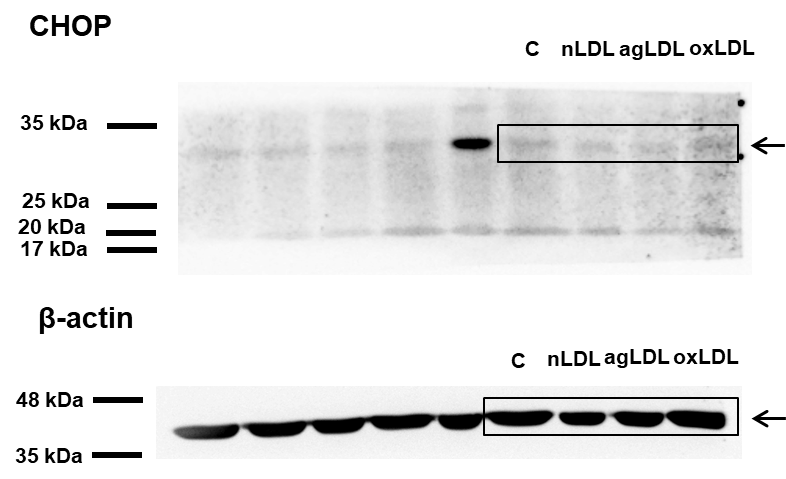


**Fig 5** raw: images acquired using ImageQuant LAS4000 imaging system and ImageQuant-TL software
